# Analysis of Gene Expression Profiles in Leaf Tissues of Cultivated Peanuts and Development of EST-SSR Markers and Gene Discovery

**DOI:** 10.1155/2009/715605

**Published:** 2009-06-24

**Authors:** Baozhu Guo, Xiaoping Chen, Yanbin Hong, Xuanqiang Liang, Phat Dang, Tim Brenneman, Corley Holbrook, Albert Culbreath

**Affiliations:** ^1^Crop Protection and Management Research Unit, USDA-ARS, Tifton, GA 31793, USA; ^2^Department of Plant Pathology, University of Georgia, Tifton, GA 31793, USA; ^3^Crops Research Institute, Guangdong Academy of Agricultural Sciences, Guangzhou, 51064 China; ^4^National Peanut Research Laboratory, USDA-ARS, Dawson, GA 31742, USA; ^5^Crop Genetics and Breeding Research Unit, USDA-ARS, Tifton, GA 31793, USA

## Abstract

Peanut is vulnerable to a range of foliar diseases such as spotted wilt caused by *Tomato spotted wilt virus* (TSWV), early (*Cercospora arachidicola*) and late (*Cercosporidium personatum*) leaf spots, southern stem rot (*Sclerotium rolfsii*), and sclerotinia blight (*Sclerotinia minor*). In this study, we report the generation of 17,376 peanut expressed sequence tags (ESTs) from leaf tissues of a peanut cultivar (Tifrunner, resistant to TSWV and leaf spots) and a breeding line (GT-C20, susceptible to TSWV and leaf spots). After trimming vector and discarding low quality sequences, a total of 14,432 high-quality ESTs were selected for further analysis and deposition to GenBank. Sequence clustering resulted in 6,888 unique ESTs composed of 1,703 tentative consensus (TCs) sequences and 5185 singletons. A large number of ESTs (5717) representing genes of unknown functions were also identified. Among the unique sequences, there were 856 EST-SSRs identified. A total of 290 new EST-based SSR markers were developed and examined for amplification and polymorphism in cultivated peanut and wild species. Resequencing information of selected amplified alleles revealed that allelic diversity could be attributed mainly to differences in repeat type and length in the SSR regions. In addition, a few additional INDEL mutations and substitutions were observed in the regions flanking the microsatellite regions. In addition, some defense-related transcripts were also identified, such as putative oxalate oxidase (EU024476) and NBS-LRR domains. EST data in this study have provided a new source of information for gene discovery and development of SSR markers in cultivated peanut. A total of 16931 ESTs have been deposited to the NCBI GenBank database with accession numbers ES751523 to ES768453.

## 1. Introduction

Peanut (*Arachis hypogaea * L.), an important source of oil, protein, and other nutrients worldwide, is ranked as the second most important seed legume after soybean. In recent years emphasis has been placed on the improvement of cultivated peanuts and the development of new cultivars with higher levels of resistance to fungal and viral diseases, which have caused reduced levels of peanut production worldwide [1–3]. *Tomato spotted wilt virus * (TSWV), a member of the genus *Tospoviruses*, causes significant yield loss in many production areas. Additionally, both early leaf spot (*Cercospora arachidicola*) and late leaf spot (*Cercosporidium personatum*) are also severe and widespread diseases of peanut. Standard control methods remain a limitation since severity of the disease may not correspond to crop rotation and/or other field treatment practices. Therefore, the improvement of existing cultivars and/or development of new cultivars with greater levels of field resistance is the most effective economical means of controlling these diseases and is a major objective in peanut breeding programs [4]. Although peanut cultivars and breeding lines with greater resistance to TSWV or leaf spots have been developed and released [4, 5], variations in disease resistance among these cultivars do exist. To supplement this incomplete resistance, single or multiple genes with known metabolic pathway(s) can be engineered into existing peanut cultivars to increase the effectiveness of natural disease resistance. These so-called “enhanced” peanuts could be bred with conventional peanut cultivars to maintain high plant fitness and yield [1, 2].

Previously, some laboratories have used genetic engineering to develop new peanut genotypes with disease resistance, usually transferring resistance gene(s) from other plant species into peanut [1, 2, 6, 7]. This approach typically targets a single gene, which may not be able to provide adequate levels of resistance, and may easily be overcome by the pathogen. Genetics research indicates that peanuts may have evolved a series of defense mechanisms against invasion by plant pathogens [8]. This suggests that peanut ESTs (expressed sequence tags) from disease resistant genotypes may be an asset to discover native defense/resistance genes. Generating sequences from cDNA fragments can be used to discover new genes and to assess their expression levels in the representative tissues. In addition, the availability of cDNA sequences has accelerated further molecular characterization of genes of interest and provided sequence information for marker development, microarray construction, and genome annotation. The availability of this resource may enable the identification and analysis of complex biological interactions between plant and pathogens. Model organisms such as *Arabidopsis thaliana * and rice were selected for genome sequencing because of the relatively small genome size. Given the complexity and large size of the cultivated peanut genome (2*n* = 4*x* = 40 and 2800 Mb/1C), it is difficult to imagine sequencing the whole peanut genome at this point of time. Therefore, a significant insight into the functional portion of the peanut genome can be gained through large-scale production and analysis of ESTs. In peanut genome research, only a handful of studies have been conducted using this strategy for discovering resistance genes. Luo et al. [9] reported upregulated genes in response to leaf spot disease using cDNA microarray and real-time PCR. Other disease resistance genes (such as TSWV) were reported in *Lycopersicon peruvianum * [10], * L. esculentum * [11], and *Capsicum chinense * [12].

Large variations have been recorded for morphological and agronomic traits for cultivated peanut, whereas few molecular variations have been reported by using current molecular technologies such as restriction fragment length polymorphism (RFLP), random amplified polymorphisms (RAPD), amplified fragment length polymorphisms (AFLP), and simple sequence repeats (SSRs) [13–17]. With the accumulation of EST sequences in the public database, a large number of available sequences presents opportunities to electronically identify and validate usefulness of potential molecular markers (i.e., SSRs or microsatellites) at a low cost and in an efficient manner [18, 19]. Some SSRs lie within the coding region of cDNA sequences, allowing the prediction of putative functions through homology searches from different biological databases (i.e., NCBI). The SSR markers developed from EST sequences, with putative biological functions, can be evaluated for association with phenotypes [20].

In order to increase gene diversity in the EST collection and to enhance the probability of identifying genes associated with disease resistance, the libraries were prepared from leaf tissues of two different cultivated peanut genotypes under the same field conditions. A total of 17 376 ESTs were sequenced, resulting in 6,888 unique EST sequences. A variety of computational approaches were employed to conduct an extensive analysis of these EST sequences to identify novel defense-related genes and new potential molecular markers. A total of 290 new EST-based SSR markers were developed (see Table S1 in Supplementary Material available online at doi: 10.1155/2009/715605) and some defense-related transcripts were also identified, such as putative oxalate oxidase (EU024476) [21], putative TSWV resistance gene [22], and NBS-LRR domains.

## 2. Materials and Methods

### 2.1. Libraries Construction and Sequencing

Leaf tissues were collected at 100 days after planting (DAP) under the natural occurrence of spotted wilt and leaf spot diseases of peanut genotypes, Tifrunner [23], GT-C20, and A13 [9, 24]. Tissues were quickly frozen in liquid nitrogen and stored at −80°C until RNA extraction. Tifrunner is resistant to TSWV and leaf spots but susceptible to *Aspergillus flavus*. GT-C20 is susceptible to TSWV and leaf spots but resistant to *A. flavus*, and A13 (NCV11 × AR4) is moderately resistant to TSWV and leaf spots, and resistant to *A. flavus * infection [25].

The procedures for constructing cDNA libraries from leaf tissue were performed as reported previously [9]. The two libraries, C20L and TFL, were named after source genotypes GT-C20 and Tifrunner, respectively, and cDNA libraries were also constructed for A13 (where only a little over 2 000 ESTs sequenced and batch released without further discussion). After the quality of each library was assessed, sequencing reactions were performed using ABI 3730XL Genetic analyzer (Applied Biosystems) with the ABI Prism BigDye terminator cycle sequencing kit v3.0 (Foster City, Calif, USA) from 5′ end of cDNA with T3 (cDNA ligated to the pT7T3 vector) sequencing primer.

### 2.2. EST Processing and Clustering

The cDNA sequences were analyzed with Sequencher v4.6 (Gene Codes, Ann Arbor, Mich, USA). Vector and low quality sequences were removed. The remaining small sequences (less than 100 nucleotides) were also removed. Resulting high-quality cDNA sequences were separately assembled into contigs through the use of TGICL program (Pertea et al., 2003). The criteria for clustering are sequence sharing greater than 90% identity over 40 or more contiguous bases with unmatched overhang less than 30 bases in length. Overlaps exclusively on low complexity regions were excluded.

### 2.3. Functional Annotation of Unique ESTs and Bioinformatics Analysis

In order to identify the putative function of unique ESTs based on the homology, the nonredundant protein (nr) database at the NCBI (National Center for Biotechnology Information) GenBank was downloaded and localized. The unique EST sequences obtained in this study were BLASTed (BLASTx) [26] against the nr database. The unique EST sequences were considered to be homologous to known proteins in nr database when the *E*-value of BLAST was less than 1e^−5^ and the BLAST score was higher than 100. Resistance and defense-related genes were identified in the unique ESTs according to similarity known resistance/defense genes in the public database. The putative full-length protein-coding regions were determined by complete open reading frame (ORF), poly (A), and significant similarity to known protein sequence.

Classification of unique EST sequences was analyzed using the Munich Information Center for Protein Sequences (MIPS), Arabidopsis Sequencing Project Functional Categories [27, 28], and the Gene Ontology Consortium [29]. The unique EST sequences were BLASTed against MAtDB (MIPS *Arabidopsis thaliana * Database), and matched unique sequences were sorted into different categories according to MIPS Functional Catalogue Database. To further classify and identify the biological roles and molecular functions of the unique EST sequences, we downloaded The Institute for Genomic Research (TIGR) *Arabidopsis thaliana * gene index (AGI, Release 13.0), soybean gene index (GMGI, release 12.0), and *Medicago truncatula * gene index (MTGI, Release 8.0). BLAST program was used to compare unique EST sequences to the Tentative Consensus (TC) sequences with terms from Gene Ontology Consortium controlled vocabularies. The expectation value cutoff for BLAST was set at 1e^−5^.

To analyze relationships between our EST sequences and other plant ESTs, we downloaded TIGR *Arabidopsis thaliana * gene index release 13.0 (June 16, 2006) consisting of 81 826 unique sequences, rapeseed (*Brassica napus*) gene index 2.0 (June 16, 2006) consisting of 25 929 unique sequences, maize (*Zea mays*) gene index 17.0 (November 14 2006) consisting of 115 744 unique sequences, *Medicago truncatula * gene index 8.0 (January 19, 2005) consisting of 36 878 unique sequences, rice (*Oryza sativa*) gene index 17.0 (June 20, 2006) consisting of 181 796 unique sequences, soybean (*Glycine max*) gene index 12.0 (September 20, 2004) consisting of 63 676 unique sequences, and wheat (*Triticum aestivum*) gene index 10.0 (January 14, 2005) consisting of 122 282 unique sequences (ftp://occams.dfci.harvard.edu/pub/bio/tgi/data/). A sequence similarity comparison between the Tentative Consensus (TC) sequences of these Gene Indices and our EST sequences was performed using the BLASTn algorithm, with 80% or 90% identity and a 1e^−5^ *e*-value as the cutoff values.

### 2.4. Characterization of Newly Developed SSR Markers

After trimming and assembling the EST sequences, a Perl script known as MIcroSAtellite (MISA http://pgrc.ipk-gatersleben.de/misa/) was used to identify microsatellites in the unique ESTs. In this study, EST-based SSRs were considered to contain motifs one to six nucleotides in size with five or more motif repeats. Frequency of EST-SSR refers to kilo-base pairs of EST sequences containing one SSR. As a result, we developed 290 new SSRs (Supplementary Table S1) and tested these SSRs against a set of diverse peanut accessions, including cultivated and wild species for amplification and polymorphisms. PCR products amplified by SSR primer pair EM-31 were cloned and sequenced for confirmation and comparison of simple sequence repeats among several peanut accessions.

## 3. Results and Discussion

### 3.1. Generation of ESTs Derived from Peanut Leaf cDNA Libraries

A total of over 20 000 EST sequences were generated, including 17 376 ESTs from TFL and C20L, and subjected to quality analysis using Sequencher software. After trimming vector and discarding low-quality sequences from the raw sequences, 16 931 high-quality EST sequences (over 80%) were obtained for further analysis (sequences smaller than 100 bp were excluded). These included 8328 sequences derived from GT-C20 and 6104 from Tifrunner. The percentages of acceptable quality EST sequences for C20L and TFL were 89% and 76%, respectively. In GT-C20, approximately 5.04 Mb of peanut sequences were generated with insert sizes ranging from 138 bp to 999 bp, averaging 541 bp per sequence read. In Tifrunner, approximately 3.03 Mb of peanut sequences were produced with an average length of 375 bp per EST (ranging from 137 to 1191 bp). In order to reduce the redundancy and produce longer consensus sequences, EST sequences were assembled within each genotype. This resulted in a total of 6888 unique EST contigs, out of which 3976 were from GT-C20 and 2912 came from Tifrunner. Seventy-five percent of total unique sequences were comprised of the singletons and only 53 (about 3%) of all contigs contained more than twenty members, with 1650 (about 97%) consisting of 2 to 20 members. The percentage of redundancy in both libraries was about 52%.

### 3.2. Overlapping of Peanut Genes and High Expression Genes in Resistant and Susceptible Genotypes

A comparative analysis of common and unique sets of expressed genes between resistant and susceptible genotypes may improve our understanding of which genes may be associated with defense response to TSWV or leaf spot. The unique sequences in C20L and TFL, having at least 40 bases with >90% identity and less than 20 mismatches, were identified as a part of the same consensus transcript. When comparing the ESTs from the resistant genotype Tifrunner library to the ESTs from the susceptible genotype GT-C20 library, only 948 (about 14%) of ESTs were present in both libraries. The remaining 3028 in C20L and 1964 in TFL were shown to be library specific. These results indicated that the relative gene expression profiles between GT-C20 and Tifrunner were significantly different, possibly indicating the relative importance of specific gene transcripts to the levels of disease resistance.

Highly expressed genes were identified by counting the number of ESTs/clones in a certain contig in each of the libraries. The top 40 highest redundant genes with putative associated functions (BLASTx search against NCBI nr database) in C20L and TFL libraries were counted and compared (Tables 1  and 2). Resistance/defense-related genes found in the resistant genotype (Tifrunner) were metallothionein-like protein (TFLcontig5) and heat shock protein Hsp20 (TFLcontig34). Plastic aldolase (C20Lcontig36) and glycolate oxidase (C20Lcontig97) were present in the susceptible genotype (GT-C20). Catalase (C20Lcontig127 and TFLcontig87) was present in both libraries. Highly-expressed genes present in both genotypes involved in photosynthesis were expected. Interestingly, several virus genes were identified in the two libraries, such as polyproteins from peanut mottle virus and bean common mosaic virus strain peanut stripe. The presence of these viral transcripts suggests that these viruses were present in peanut leaf tissues.

### 3.3. Functional Classification of Unique EST Sequences

**Figure 1:**
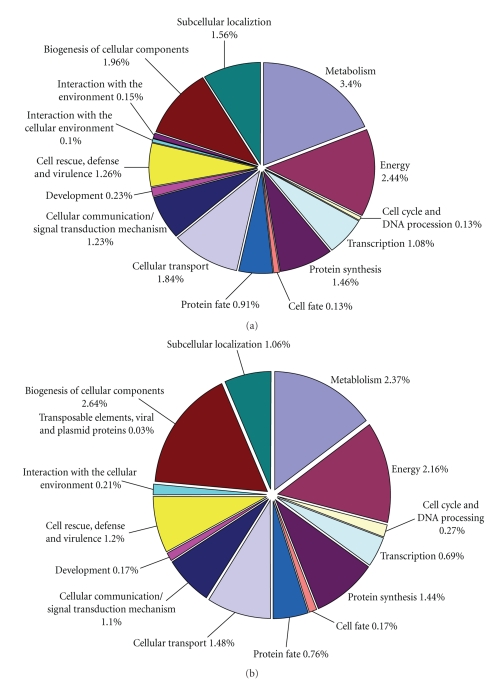
Functional classification of peanut unique EST sequences by referring to Arabidopsis Sequencing Project Functional Categories. The matched unique sequences are shown in figures. The unique sequences which have no significant homology to *Arabidopsis* genes or matched to those that are unknown and unclassified genes were described in text. (a) Functional categories of GT-C20 unique EST sequences. (b) Functional categories of Tifrunner unique EST sequences.

In order to characterize the putative functions of unique sequences and involvement in different biological processes, a similarity search against MIPS *Arabidopsis thaliana * Database [27, 28] was performed using BLASTx algorithm. Surprisingly, 82% ( 3265) of GT-C20 unique EST sequences and 84% ( 2452) of Tifrunner unique EST sequences have no putative functions. These unique sequences were comprised of some electronic translated proteins with no significant homologies to *Arabidopsis * proteins and some matched to *Arabidopsis * proteins but did not have assigned biological functions. The remaining unique sequences with significant similarity (less than 1e^−5^ as a cutoff value) to *Arabidopsis * proteins were sorted into fifteen and fourteen categories for GT-C20 and Tifrunner, respectively (Figure 1). The largest proportion of genes was found to participate in the biological process of metabolism (3.4% in GT-C20 and 2.37% in Tifrunner). The Energy category (2.44% in GT-C20 and 2.16% in Tifrunner) was ranked second since leaf tissues were used in the construction of cDNA libraries. Defense-related genes were 1.26% in GT-C20 and 1.2% in Tifrunner, and environmental-interacting genes were 0.25% in GT-C20 and 0.2% in Tifrunner.

Given that the MIPS functional category system is based on one model species (i.e., *Arabidopsis*) representing only a small portion of all genes in plants, many peanut EST sequences that might match to known genes in other plants cannot be assessed. To further identify and categorize biological and molecular functions of unique EST sequences, we used another classification system, the Gene Ontology (GO) for annotation of these ESTs. The BLAST program was employed to analyze gene ontology assignments against TIGR gene indices including soybean, *Medicago truncatula * and *Arabidopsis*. In total, 3443 unique EST sequences in C20L and TFL libraries were classified into three broad categories, “biological process,” “cellular component” and “molecular function,” with 7913 GO functional terms (Table 3). Since any given unique sequence may be assigned to more than one GO functional terms and one “child” term can fit into multiple parental categories, the total number of GO mappings in each of the three broad categories will be beyond the actual number of unique sequences.

In the C20L library, 2109 unique EST sequences containing 1461 singletons and 648 contigs were assigned to 4764 GO functional terms. Of these sequence, 1588 (75.3%), 1993 (94.5%), and 1401 (66.4%) were assigned to biological processes, molecular functions, and cellular components, respectively. In the biological processes category, a large proportion of unique genes were observed to involve cellular processes (56.7%); the metabolic processes (16.3%) and the biological regulations (13.5%) ranked second and third. It is worthy to note that approximately 12% of the unique sequences correspond to potential responsive proteins of various stimuli, which in turn, could be further divided into eight smaller categories including response to different stresses (16 unique sequences), biotic stimuli (14 unique sequences), abiotic stimuli (62 unique sequences), and defense responses (11 unique sequences). Within the broad category of molecular functions, the three most dominant smaller categories were catalytic activity (43.95%), binding (30.4%), and structural molecular activity (7.4%).

In the TFL library, 1334 unique EST sequences consisted of 877 singletons and 457 contigs were classified into biological processes, molecular functions, and cellular components, accounting for approximately 75.4%, 92.9%, and 70.5% of the 1334 unique sequences (corresponding to 3149 GO functional terms), respectively. The total number of GO terms associated with biological processes was 1006, which could be further divided into 15 smaller categories. The three most dominant categories of unique EST sequences in biological processes were cellular processes (54.7%), metabolic processes (20.2%), and response to stimuli (17.0%). Within the response to stimuli category, we further classified 11 unique sequences in term of response to stresses, 20 unique sequences in term of response to biotic stimuli, 82 unique sequences in term of response to abiotic stimuli, and 9 unique sequences in term of defense responses. The GO assignment also yielded 1239 and 940 unique sequences associated with molecular functions and cellular components, respectively. The molecular functions category could be further divided into 9 smaller categories. Of these, a large proportion of unique sequences were found to be related to catalytic activities (37.6%), followed by binding (35.6%).

### 3.4. Development of EST-Derived SSR Markers

As previously observed, vast variations have been recorded for morphological and agronomic traits in cultivated peanut, whereas few molecular variations and low genetic diversity have been reported [13–17]. The EST sequences generated in this study were used to detect possible microsatellites which contain di- to hexanucleotide SSR with a minimum of five repetitions of all motifs via the MISA Perl script (http://pgrc.ipk-gatersleben.de/misa/).

In the original SSR search in all unassembled EST sequences, 8328 GT-C20 EST sequences and 6104 Tifrunner EST sequences were examined. A total of 682 EST sequences in GT-C20 and 323 EST sequences in Tifrunner were found to contain microsatellites. These numbers correspond to 8.2% and 5.3% of total EST sequences of GT-C20 and Tifrunner, respectively. After clustering and assembly of the two libraries separately, the microsatellite search was conducted again, and the number of SSR-containing EST sequences was reduced to 565 in GT-C20 (246 contigs and 319 singletons) and 245 in Tifrunner (84 contigs and 161 singletons). A reduction of 9.2% occurred in GT-C20 while a sharp reduction of 24% was observed in Tifrunner. The assembly of EST sequences with the two genotypes resulted in a nonredundant set of 593 SSRs in GT-C20 and 263 SSRs in Tifrunner. In GT-C20, 3976 unique EST sequences were surveyed for a total of 2.68 million base pairs (Mbps). In Tifrunner, 2912 unique EST sequences were surveyed for a total of 1.55 Mbp. The compilation of all SSRs revealed that, on the average, one SSR can be found every 4.52 kb in GT-C20 ESTs; while in Tifrunner one SSR was found in every 5.89 kb.

**Figure 2:**
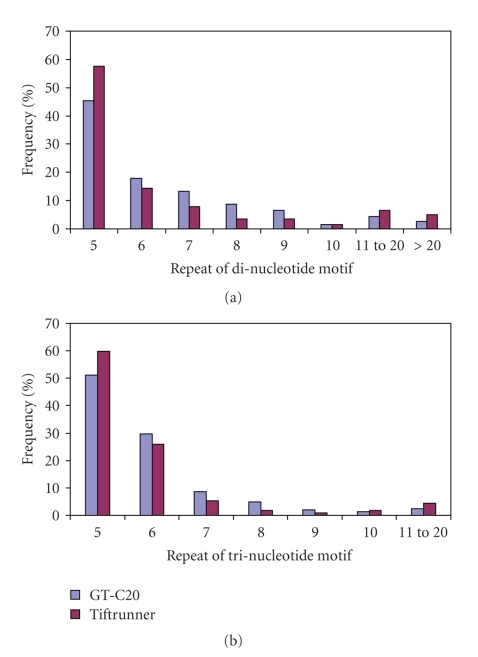
Distribution of repeat units in dinucleotide motif and trinucleotide motif in ESTs from GT-C20 and Tifrunner. Y-axis represents the frequency of microsatellites of a specific motif repeat. X-axis represents the number of repeats for (a) dinucleotide motif and (b) trinucleotide motif.

Among the 593 SSRs in GT-C20, the dinucleotide repeat motif was the most abundant type of SSRs (59.5%), followed by tri- (33.7%), compound (4.5%), tetra- (1.6%), hexa- (0.3%) and pentanucleotide (0.2%) repeat motifs. A similar trend in repeat motif distribution was found in Tifrunner. The dinucleotide repeat motif showed a higher frequency in GT-C20 unique EST sequences than in Tifrunner, while other repeat motifs (tri- to hexanucleotide motifs) were lower in GT-C20 than in Tifrunner. Dinucleotide and trinucleotide repeat motifs were further analyzed for SSR length (or number of repeat units). There were similar distribution profiles of dinucleotide and trinucleotide motifs in GT-C20 and Tifrunner. Within the dinucleotide motif, the frequencies of five, eleven to twenty and more than twenty repeating units were higher in Tifrunner than in GT-C20 (Figure 2(a)). Similar results were found in trinucleotide motif (Figure 2(b)).

**Figure 3:**
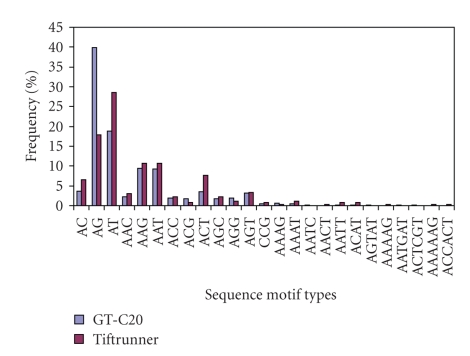
Distribution of peanut leaf EST-derived SSRs according to motif sequence type. X-axis is motif sequence types (considering sequence complementary), and Y-axis represents the frequency of SSRs of a given motif sequencer type.

Within the three dinucleotide repeat types, the AG dinucleotide repeat motif was the most abundant motif detected in GT-C20 (39.97% considering sequence complementary), followed by the motif AT (18.89%), while the AT was most abundant in Tifrunner (28.52% considering sequence complementary) and the AG was the second most common motif recovered in Tifrunner (17.87%) (Figure 3). The AC dinucleotide repeat motif was the least motif found in both GT-C20 and Tifrunner. All ten trinucleotide repeat motif types were found in both GT-C20 and Tifrunner (Figure 3). The most abundant trinucleotide motifs in both genotypes were AAG and AAC with overall frequencies of 9.44% in GT-C20 and 10.65% in Tifrunner. Both genotypes shared ACT as the second most abundant trinucleotide repeat motif (3.54% in GT-C20 and 7.60% in Tifrunner). The least abundant repeat motif in GT-C20 was CCG motif with frequency of 0.51%, while in Tifrunner the least abundant repeat motifs were CCG and ACG (each 0.76%). Five tetranucleotide repeat motifs were detected both in GT-C20 and Tifrunner. Of these the AACT repeat motif was not found in GT-C20 while the AATC repeat motif did not appear in Tifrunner. For tetranucleotide repeat, the most dominant repeat motif in GT-C20 was AAAG (0.67%), while in Tifrunner, it was AAAT (1.14%). Interestingly, the pentanucleotide and hexanucleotide repeat motifs detected in GT-C20 were completely different from those in Tifrunner. The pentanucleotide (AGTAT) and hexanucleotide (AATGAT and ACTCGT) motifs were present in GT-C20 while absent in Tifrunner, whereas the AAAAG, AAAAAG, and ACCACT motifs were not observed in GT-C20 but in Tifrunner.

### 3.5. Putative Gene Discovery

Plants, naturally exposed to different pathogens and various environmental conditions, have evolved different defense mechanisms. One type of defense mechanisms involves the specific recognition of pathogens by plants [30]. A class of resistance genes (named R genes) has been identified in plant-pathogen recognition and response [31]. The R gene products (R proteins) can be divided into different families based on their domain composition, the so-called NBS-LRR (containing both a nucleotide binding site domain and leucine-rich repeats) represents the largest class of R proteins [32]. In this study, ten unique EST sequences from GT-C20 library and 9 unique EST sequences from Tifrunner had high homologies to known genes containing NBS-LRR domain. The LRR domain is involved in the regulation of signaling activity of the R protein and a single amino acid change in this domain can result in a prolonged activation of this protein [33, 34]. In plants, a mechanism of resistance against pathogen infection in several R proteins involved the activation of molecular chaperones or heat shock proteins (HSPs) [35–38]. Rapid expression of heat shock proteins (HSPs) was also observed to be a common plant response to a variety of stress factors [35]. In the unique EST sequences, twenty five of GT-C20 and twenty eight of Tifrunner sequences had significant homology to HSP genes.

Another stress-induced transcript was observed to be abundant in peanut ESTs. Seventy six unique sequences from the two libraries were found to match methallothionein or metallothionein-like genes. Metallothioneins are a superfamily of ubiquitously expressed, low molecular mass (6-7 kD), cysteine-rich proteins that have a high binding affinity to bivalent metal ions. Metallothioneins are known to be involved in metal detoxification, homeostasis, and protection against oxidative damage [39]. These proteins were first discovered in animals and now have been found in virtually all organisms including plants, fungi, and some prokaryotes [40, 41]. Previous studies in peanut showed that metallothionein transcripts were present in both cultivated peanut and wild species [24, 42].

We have identified and cloned one peanut endogenous germin-like/oxalate oxidase gene named *AhOxOl * (EU024476) [21], originating from peanut leaf cDNA libraries. The *AhOxOl * including 991 bp cDNA sequence encodes a 219 amino acid protein with a 21-residue signal peptide. After cleavage of the signal peptide, it has a mass of 20.84 kDa. This protein contains three motifs, Q/NDL/FCVAD, G(X)5HXH(X) 11G, and G(X)5P(X) 4H(X) 3N, which are characteristic to germin-like proteins. Furthermore, the deduced protein of *AhOxOl * consists of the “germin box” (HI/THPRATEI), which is a conserved sequence shared by germins within the motif G(X)5HXH(X) 11G. Research has suggested the enhancement of resistance to *Sclerotinia minor * in peanut by expressing a barley oxalate oxidase gene [7]. Oxalate oxidase belongs to the germin family of proteins and acts as a source of hydrogen peroxide (H_2_O_2_) in certain plant-pathogen interactions. We also identified a putative TSWV resistance gene [22] from these EST sequences, which is under further investigation.

### 3.6. Comparison of EST Data to Other Plant Sequences

In order to investigate how many of these peanut ESTs were homologous to plant transcripts in other publicly available plant EST databases, a comparative analysis of peanut ESTs to several plant EST databases, such as soybean, *Medicago truncatula*, *Arabidopsis*, rapeseed, rice, maize, and wheat TIGR gene indices, was performed (Table 4). When the cutoff value of sequence identity was more than 80%, the percentage of peanut EST sequences matching soybean and *Medicago truncatula * was approximately 49.78% and 39.55% (Table 4), respectively. Once the cutoff value increased to more than 90%, the percentage of peanut EST sequences matched to soybean and *Medicago truncatula * sharply drops to approximate 3.76% (a reduction of 46.02%) and 2% (a reduction of 39.55%), respectively. When DNA sequence identity was set more than 80%, the percentages of peanut EST sequences matching *Arabidopsis*, rapeseed, rice, maize, and wheat were 12.24%, 9.03%, 12.095, 10.39%, and 9.79%. When DNA sequence identity was at ≥90%, there was no significant difference found among these species, except that rapeseed had the least percentage (0.54%). These results indicated that when DNA sequence identity was at ≥80%, peanut EST sequences showed higher homology to EST sequences of legume species than to those of other plants including cereal species and dicot plants.

### 3.7. Characterization of Newly Developed SSR Markers

There were 593 and 263 SSRs detected in GT-C20 and Tifrunner nonredundant sequences, respectively. Together, we collected 780 SSR-containing sequences; and 490 sequences did not qualify for primer design as the flanking sequences were too short or too poor in quality. Primers were designed for remaining 290 SSR-containing sequences (Supplementary Table S1). Of the 290 designed EST-SSRs, 65 SSRs were found in the 5′ untranslated regions (5′ UTR), 178 in coding regions, and 47 in the 3′ UTR. Among the 290 primer pairs, 251 primer pairs were successful in PCR amplification in cultivated and wild peanuts tested in this study. The other 39 primers failed to amplify at various annealing temperatures and Mg2+ concentrations and were excluded from further analysis. Among the 251 working primer pairs, 182 amplified PCR products at the expected sizes, and 41 primer pairs resulted in larger PCR products than what expected, suggesting that there may be an intron within the amplicons. The amplified products of the other 28 primer pairs were smaller than expected size, suggesting the occurrence of deletion within the genomic sequences or a lack of specificity. 

**Figure 4:**
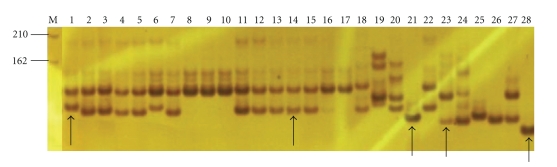
Amplification patterns obtained with primer EM-31 in polyacrylamide gel electrophoresis of cultivated peanuts, including Chinese landrace and breeding lines, and US market types of runner, Spanish and Virginia, and wild species. Arrows indicate the bands excised for sequencing. M = molecular weight marker in base pair, 1 = Guangliu, 2 = Sanyuening, 3 = GT-C20, 4 = Spancross, 5 = Tennessee Red, 6 = Xiaoliuqiu, 7 = Yangjiangpudizan, 8 = Xihuagoudo, 9 = Padou, 10 = Bo-50, 11 = Yingdejidouzai, 12 = Heyuanbanman, 13 = Tosunxiaohuasheng, 14 = SunOleic 97R, 15 = Tifrunner, 16 = Georgia Green, 17 = NC94022, 18 = *A. villosa*, 19 = *A. stenosperma*, 20 = *A. correntina*, 21 = *A. cardenasii*, 22 = *A. magna*, 23 = *A. duranensis*, 24 = *A. chacoensis*, 25 = *A. batizocoi*, 26 = *A. helodes*, 27 = *A. monticola*, 28 = *A. pintoi*.

**Figure 5:**
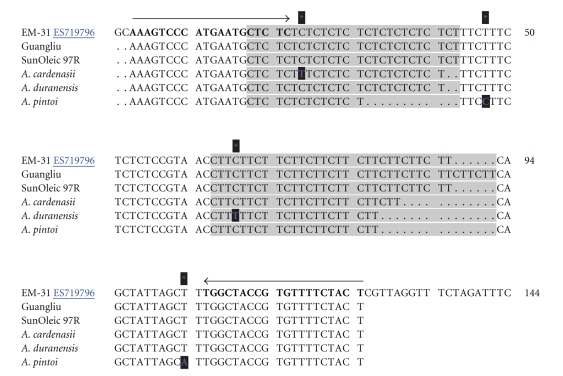
Sequences comparison. SSR primer EM-31 amplificon sequences were obtained as indicated in Figure 4. The original EST sequence (ES719796) was included, where SSR primer EM-31 was designed. The pair of forward and reverse primer sequences was indicated by a pair of lines with arrows and bold-faced. The repetitive sequence regions were shaded in gray. The single nucleotide polymorphisms were indicated by dark-shaded and purple-colored star.

Within cultivated peanuts, 26 EST-SSRs exhibited polymorphism. For the wild species, 221 primer pairs (88%) were polymorphic. In order to confirm how SSR polymorphisms are produced, the amplified products of 4 cultivated peanuts and 3 wild species by SSR marker EM-31 were cloned and sequenced (Figures 4  and 5). All the sequenced alleles from both cultivars and wild species were highly identical to the original EST sequence (ES719796) from which the EST-SSR marker was designed. The alignment of the sequences of the amplicons showed that all the primer-binding regions are conservative. The allelic diversity could be attributed mainly to differences in repeat number in the microsatellite regions. Additional substitutions were also observed in the regions flanking the microsatellite regions. Out of the four single base (SNP) point mutations, there was one in * A. cardenasii*, one in *A. durangensis*, and two in *A. pintoi*. The comparison of these sequences of cross-species amplicons further confirmed the conservation and transferability of the developed EST-SSRs. Thus, these markers will provide a valuable resource for genetic linkage mapping, QTL analysis, and marker-assisted selection.

The average percentage of polymorphism of EST-based SSRs was 9.96% in cultivated peanuts tested in this study. This value was lower than those of genomic SSR found in other studies [43–45], but was higher than that tested by using RAPD (6.6%) [46], and AFLP (6.7%) [14].

## 4. Conclusion

The collection of cultivated peanut leaf EST sequences in this study provides a valuable public genomic resource for the development of genomic tools and functional genomics studies and will improve the understanding of peanut defense-mechanism(s) to various diseases (TSWV and leaf spots). The new set of EST sequences is an important addition to publicly available resources, especially in relation to the study of biotic stresses in peanut. We have identified potential disease-resistance genes and have provided a list of putative functional features that can aid in the understanding of how gene expression may be involved in different biological processes. Additionally, this study demonstrated that large-scale EST sequencing efforts can lead to an identification of potential molecular markers which may help to accelerate traditional breeding processes and linkage map development [47]. In summary, large number of peanut EST sequences and the related annotation information will provide an important resource of sequences and information for the peanut community. This in turn will aid in the discovery of novel genes and will stimulate the development of new molecular markers for future peanut research. Progress is underway to construct peanut oligo microarray and develop cultivated peanut genetic linkage mapping populations for linkage map and QTL studies by using the uniESTs and SSRs.

## Supplementary Material

Supplementary Table S1 shows a total of 290 new developed EST-based SSR markers and some identified defense-related transcripts, such as putative oxalate oxidase (EU024476), putative TSWV resistance gene, and NBS-LRR domains.

## Figures and Tables

**Table 1:** Similarity BLAST search against nr database for GT-C20 top 40 abundant contigs.

Contig	No. of clones	Accession no.	Planta	Putative genes description	*E*-value
C20Lcontig37	455	gb|AAG24882.1|	Glycine. max	Ribulose-1,5-bisphosphate carboxylase small subunit rbcS1	6 e^−82^
C20Lcontig40	303	ref|NP_068348.2|	Peanut mottle virus	Polyprotein	0
C20Lcontig77	184	gb|AAB01025.1|	Bean common mosaic virus strain peanut stripe	Polyprotein	0
C20Lcontig20	143	gb|AAG61120.1|	Gossypium hirsutum	Ribulose-1,5-bisphosphate carboxylase/oxygenase activase 1	0
C20Lcontig83	81	gb|AAL29886.1|	Glycine max	Chlorophyll a/b binding protein type II	1e^−148^
C20Lcontig76	47	gb|ABC46708.1|	Arachis hypogaea	Chloroplast photosystem II 10 kDa protein	9 e^−68^
C20Lcontig86	46	gb|AAD27876.2|	Vigna radiata	Carbonic anhydrase	1e^−153^
C20Lcontig4	45	gb|ABE90224.1|	Medicago truncatula	Blue (type 1) copper domain; O-methyltransferase, family 2	1e^−178^
C20Lcontig54	41	pir||S04125|	Solanum lycopersicum	Chlorophyll a/b-binding protein type III precursor—tomato	1e^−137^
C20Lcontig8	40	gb|AAA50172.1|		Photosystem II type I chlorophyll a/b-binding protein	1e^−146^
C20Lcontig36	36	gb|AAR10885.1|	Trifolium pratense	Plastidic aldolase	0
C20Lcontig127	34	emb|CAD42908.1|	Prunus persica	Catalase	1e^−174^
C20Lcontig74	33	sp|Q02060|	Spinacia oleracea	Photosystem II 22 kDa protein, chloroplast Precursor (CP22)	1e^−111^
C20Lcontig33	32	gb|ABE83482.1|	Medicago truncatula	AAA ATPase, central region; Homeodomain-like	0
C20Lcontig70	31	emb|CAA43590.1|	Solanum lycopersicum	Type I (26 kD) CP29 polypeptide	1e^−138^
C20Lcontig97	31	prf||1803516A	Lens culinaris	Glycolate oxidase	0
C20Lcontig94	30	sp|P16059|	Pisum sativum	Oxygen-evolving enhancer protein 2, chloroplast precursor (OEE2)	1e^−115^
C20Lcontig41	29	gb|AAZ20283.1|	Arachis hypogaea	Nucleoside diphosphate kinase I	6 e^−54^
C20Lcontig71	29	gb|AAF89206.1|	Vigna radiata	LHCII type I chlorophyll a/b-binding protein	1e^−109^
C20Lcontig99	29	gb|AAW64931.1|	Nicotiana tabacum	Chloroplast ferredoxin I	7 e-62
C20Lcontig92	28	gb|ABE80998.1|	Medicago truncatula	Phosphoglycerate kinase	0
C20Lcontig101	28	gb|AAQ84170.1|	Pueraria montana var. lobata	Isoprene synthase	0
C20Lcontig108	25	ref|NP_192427.1|	Arabidopsis thaliana	Calcium ion binding	2 e^−84^
C20Lcontig111	25	sp|P26969|	decarboxylating	Glycine dehydrogenase [decarboxylating], mitochondrial precursor	0
C20Lcontig107	24	gb|AAO33588.1|	Arachis hypogaea	Putative extensin/nodulin protein	3 e^−93^
C20Lcontig112	24	gb|AAO33591.1|	Arachis hypogaea	Putative early light induced protein	3 e^−97^
C20Lcontig113	23	gb|ABE82236.1|	Medicago truncatula	BURP	1e^−103^
C20Lcontig114	22	sp|P10708|	Solanum lycopersicum	Chlorophyll a-b binding protein 7, chloroplast precursor (LHCI type II CAB-7)	1e^−145^
C20Lcontig16	21	gb|AAQ84168.1|	Pueraria montana var. lobata	1-deoxy-D-xylulose 5-phosphate reductoisomerase	0
C20Lcontig43	21	gb|ABA86963.1|	Glycine max	Glyceraldehyde-3-phosphate dehydrogenase A subunit	0
C20Lcontig117	20	gb|ABE80774.1|	Medicago truncatula	Chlorophyll A-B binding protein	1e^−145^
C20Lcontig12	19	dbj|BAB82452.1|	Vigna radiata	CYP1	4 e^−90^
C20Lcontig44	19	ref|NP_181539.1|	Arabidopsis thaliana	LHCB4.3; chlorophyll binding	1e^−115^
C20Lcontig104	19	sp|P27774|	Mesembryanthemum crystallinum	Phosphoribulokinase, chloroplast precursor (Phosphopentokinase) (PRKase) (PRK)	0
C20Lcontig32	18	sp|P17340|	Solanum lycopersicum	Plastocyanin, chloroplast precursor	7 e^−68^
C20Lcontig85	18	gb|AAD27877.1|	Vigna radiata	LHCII type III chlorophyll a/b binding protein	1e^−147^
C20Lcontig110	18	gb|ABE77926.1|	Medicago truncatula	Flavoprotein pyridine nucleotide cytochrome reductase	0
C20Lcontig118	18	emb|CAA45523.1|	Nicotiana tabacum	Photosystem I light-harvesting chlorophyll a/b-binding protein	1e^−117^
C20Lcontig120	18	gb|AAL47679.1|	Cucumis melo	Aminotransferase 1	0

**Table 2:** Similarity BLAST search against nr database for Tifrunner top 40 abundant contigs.

Contig	No. of clones	Accession no.	Planta	Putative gene description	*E*-value
TFLcontig11	271	ref|NP_068348.2|	Peanut mottle virus	Polyprotein	0
TFLcontig2	174	gb|AAG24882.1|	Glycine max	Ribulose-1,5-bisphosphate carboxylase small subunit	1e^−81^
TFLcontig12	162	gb|AAA50172.1|	Glycine max	Photosystem II type I chlorophyll a/b-binding protein	1e^−146^
TFLcontig14	140	gb|AAB01025.1|	Bean common mosaic virus strain peanut stripe	Polyprotein	0
TFLcontig1	116	gb|ABE90224.1|	Medicago truncatula	Blue (type 1) copper domain; O-methyltransferase, family	1e^−134^
TFLcontig5	66	gb|AAZ20291.1|	Arachis hypogaea	Metallothionein-like protein	3 e^−46^
TFLcontig19	65	sp|Q01516|	Pisum sativum	Fructose-bisphosphate aldolase 1, chloroplast precursor	1e^−151^
TFLcontig27	60	gb|ABA86963.1|	Glycine max	Glyceraldehyde-3-phosphate dehydrogenase A subunit	1e^−107^
TFLcontig8	55	sp|P24007|	Pyrus pyrifolia var. culta	Ribulose bisphosphate carboxylase small chain, chloroplast precursor (RuBisCO small subunit)	8 e^−75^
TFLcontig30	53	gb|AAG61120.1|	Gossypium hirsutum	Ribulose-1,5-bisphosphate carboxylase/oxygenase activase 1	1e^−170^
TFLcontig28	52	gb|AAL29886.1|	Glycine max	Chlorophyll a/b binding protein type II	1e^−148^
TFLcontig7	31	gb|AAQ84170.1|	Pueraria montana var. lobata	Isoprene synthase	1e^−166^
TFLcontig32	27	gb|ABF38996.1|	Pachysandra terminalis	Ribulose-1,5-bisphosphate carboxylase/oxygenase activase	1e^−119^
TFLcontig57	27	gb|AAS58469.1|	Gossypium hirsutum	Ultraviolet-B-repressible protein	6 e^−35^
TFLcontig45	26	sp|P10708|	Solanum lycopersicum	Chlorophyll a-b binding protein 7, chloroplast precursor (LHCI type II CAB-7)	1e^−145^
TFLcontig25	24	gb|AAD27877.1|	Vigna radiata	LHCII type III chlorophyll a/b binding protein	1e^−147^
TFLcontig43	24	gb|AAO33591.1|	Arachis hypogaea	Putative early light induced protein	1e^−101^
TFLcontig46	21	dbj|BAB82452.1|	Vigna radiata	CYP1	8 e^−91^
TFLcontig15	20	gb|AAO33588.1|	Arachis hypogaea	Putative extensin/nodulin protein	1e^−42^
TFLcontig40	20	sp|P16059|	Pisum sativum	Oxygen-evolving enhancer protein 2, chloroplast precursor (OEE2) (23 kDa subunit of oxygen evolving system of photosystem II)	1e^−116^
TFLcontig23	18	sp|P17340|	Solanum lycopersicum	Plastocyanin, chloroplast precursor	1e^−67^
TFLcontig56	17	pir||S04125	Solanum lycopersicum	Chlorophyll a/b-binding protein type III precursor—tomato	1e^−138^
TFLcontig55	16	emb|CAA43590.1|	Solanum lycopersicum	Type I (26 kD) CP29 polypeptide	1e^−138^
TFLcontig63	16	gb|AAP03873.1|	Nicotiana tabacum	Photosystem I reaction center subunit X psaK	6 e^−57^
TFLcontig64	15	emb|CAA45523.1|	Nicotiana tabacum	Photosystem I light-harvesting chlorophyll a/b-binding protein	1e^−116^
TFLcontig67	15	gb|AAH02118.1|	Mus musculus	Unknown (protein for MGC:6623)	1e^−85^
TFLcontig70	15	gb|ABM45856.1|	Arachis hypogaea	Cytosolic ascorbate peroxidase	1e^−142^
TFLcontig68	13	sp|P31336|	Gossypium hirsutum	Photosystem II 5 kDa protein, chloroplast precursor (PSII-T) (Light-regulated unknown 11 kDa protein)	2 e^−26^
TFLcontig76	13	gb|ABE77926.1|	Medicago truncatula	Flavoprotein pyridine nucleotide cytochrome reductase	1e^−158^
TFLcontig34	12	gb|ABD32352.1|	Medicago truncatula	Heat shock protein Hsp20	1e^−73^
TFLcontig48	12	sp|P14226|	Pisum sativum	Oxygen-evolving enhancer protein 1, chloroplast precursor (OEE1)	1e^−131^
TFLcontig58	12	gb|ABD28376.1|	Medicago truncatula	Photosystem I reaction centre, subunit XI	1e^−100^
TFLcontig65	12	gb|ABA08415.1|	Arachis hypogaea	Type 2 metallothionein	2e^−45^
TFLcontig78	12	ref|NP_181539.1|	Arabidopsis thaliana	LHCB4.3; chlorophyll	1e^−107^
TFLcontig79	12	gb|AAR12194.1|	Nicotiana benthamiana	Molecular chaperone Hsp90-2	1e^−120^
TFLcontig82	12	gb|AAW66657.1|	Picrorhiza kurrooa	Thiamine biosynthetic enzyme	1e^−117^
TFLcontig77	11	ref|NP_175963.1|	Arabidopsis thaliana	Unknown protein	8 e^−53^
TFLcontig87	11	emb|CAD42908.1|	Prunus persica	Catalase [Prunus persica]	1e^−102^
TFLcontig88	11	gb|AAW31666.1|	Ammopiptanthus mongolicus	Putative late-embryogenesis protein-like protein	7 e^−24^

**Table 3:** Statistics of GT-C20 and Tifrunner unique EST sequences assigned to GO functional categories.

	GO term	GO ID	GT-C20	Tifrunner
Biological_process	Rhythmic process	GO:0048511	9	3
	Response to stimulus	GO:0050896		
	Response to stress	GO:0006950	16	11
	Response to external stimulus	GO:0009605	12	4
	Response to endogenous stimulus	GO:0009719	2	1
	Response to chemical stimulus	GO:0042221	39	21
	Response to biotic stimulus	GO:0009607	14	20
	Response to abiotic stimulus	GO:0009628	62	82
	Defense response	GO:0006952	11	9
	Behavior	GO:0007610	34	23
	Reproduction	GO:0000003	50	28
	Multi-organism process	GO:0051704	4	3
	Multicellular organismal process	GO:0032501	22	12
	Metabolic process	GO:0008152	259	203
	Locomotion	GO:0040011	3	4
	Localization	GO:0051179	3	2
	Immune system process	GO:0002376	7	10
	Growth	GO:0040007	16	11
	Establishment of localization	GO:0051234	117	72
	Developmental process	GO:0032502	127	78
	Cellular process	GO:0009987	901	550
	Biological regulation	GO:0065007	214	128
	Biological adhesion	GO:0022610	13	6

Molecular_function	Transporter activity	GO:0005215	102	71
	Transcription regulator activity	GO:0030528	25	13
	Structural molecule activity	GO:0005198	147	102
	Motor activity	GO:0003774	9	3
	Molecular transducer activity	GO:0060089	16	14
	Enzyme regulator activity	GO:0030234	32	21
	Catalytic activity	GO:0003824	876	466
	Binding	GO:0005488	605	441
	Antioxidant activity	GO:0016209	24	18

Cellular_component	Macromolecular complex	GO:0032991	4	4
	Extracellular	GO:0005576	23	10
	Cell	GO:0005623	966	705

**Table 4:** Comparison of the peanut unique ESTs with those in soybean, Medicago, Arabidopsis, oilseed rape, rice, maize and wheat.

	Number of ESTs matched to TIGR gene indices	
*TIGR Gene Indices*	(Percentage in Parentheses)	
	Identity ≥ 80%	Identity ≥ 90%

*Glycine max*	3,429 (49.78)	259 (3.76)
*Medicago truncatula*	2,724 (39.55)	138 (2.00)
*Arabidopsis thaliana*	843 (12.24)	129 (1.87)
*Brassica napus*	622 (9.03)	37 (0.54)
*Oryza sativa*	833 (12.09)	209 (3.03)
*Zea mays*	716 (10.39)	180 (2.61)
*Triticum aestivum*	674 (9.79)	136 (1.97)
